# Analysis of Body Composition and Pain Intensity in Women with Chronic Pelvic Pain Secondary to Endometriosis

**DOI:** 10.1055/s-0040-1713912

**Published:** 2020-09-08

**Authors:** Joyce Beatriz da Silva, Maria Beatriz Ferreira Gurian, Carla Barbosa Nonino, Omero Benedito Poli-Neto, Antonio Alberto Nogueira, Francisco José Candido dos Reis, Júlio Rosa-e-Silva Silva

**Affiliations:** 1 Department of Gynecology and Obstetrics, Faculdade de Medicina de Ribeirão Preto, Universidade de São Paulo, Ribeirão Preto, Brazil

**Keywords:** chronic pelvic pain, fat percentage, pain intensity, anxiety, depression, dor pélvica crônica, percentual de gordura, intensidade da dor, ansiedade, depressão

## Abstract

**Objective** To determine the average body composition (percentage of body fat), the anthropometric markers, and the intensity of clinical pain in women with a clinical diagnosis of chronic pelvic pain (CPP) secondary to endometriosis.

**Methods** A case-control study performed with 91 women, 46 of whom with CPP secondary to endometriosis and 45 of whom with CPP secondary to other causes. They underwent an evaluation of the anthropometric parameters by means of the body mass index (BMI), the perimeters (waist, abdomen, hip), and the percentage of body fat (%BF), which were assessed on a body composition monitor by bioimpedance; the intensity of the clinical pain was evaluated using the visual analog scale (VAS), and the symptoms of anxiety and depression, using the hospital's anxiety and depression scale (HAD).

**Results** The groups did not differ in terms of mean age, BMI, %BF or regarding the available waist-to-hip ratio (WHR). The mean intensity of the clinical pain by the VAS was of 7.2 ± 2.06 in the group with CPP secondary to endometriosis, and of 5.93 ± 2.64 in the group with CPP secondary to other causes ( *p* = 0.03), revealing significant differences between the groups.

**Conclusion** We concluded that, despite the difference in the pain score assessed between the two groups, there was no difference regarding body composition and anthropometry.

## Resumo

**Objetivo** Determinar a composição corporal média (porcentagem de gordura corporal), os marcadores antropométricos, e a intensidade de dor clínica em mulheres com diagnóstico clínico de dor pélvica crônica (DPC) secundária a endometriose.

**Métodos** Um estudo de caso-controle realizado com 91 mulheres, 46 das quais com DPC secundária a endometriose, e 45 das quais com DPC secundária a outras causas. As pacientes foram submetidas à avaliação dos parâmetros antropométricos por meio do índice de massa corporal (IMC), dos perímetros (cintura, abdômen, quadril), e do percentual de gordura corporal (%GC), que foram avaliados em monitor de composição corporal por bioimpedância; a intensidade clínica da dor foi avaliada usando-se a escala visual analógica (EVA), e os sintomas de ansiedade e depressão, usando a escala de ansiedade e depressão do hospital (EADH).

**Resultados** Os grupos não diferiram quanto à idade média, ao IMC, ao %GC, nem quanto à relação da cintura–quadril (RCQ) disponível. A média da intensidade clínica da dor pela EVA foi de 7,2 ± 2,06 no grupo com DPC secundária a endometriose, e de 5,93 ± 2,64 no grupo com DPC secundária a outras causas ( *p* = 0,03), revelando diferenças significativas entre os grupos. Em relação à EADH, ambos os grupos estavam acima da média de corte.

**Conclusão** Concluímos que, apesar da diferença no escore de dor avaliado entre os dois grupos, não houve diferença com relação à composição corporal e à antropometria.

## Introduction

Chronic pelvic pain (CPP) is a frequent complaint in the gynecological practice, and it causes suffering, compromises the quality of life of the woman, and results in high costs to health systems. 1 The prevalence of CPP is not well established, and it may vary from one country to another. However, it is estimated that 3.8% of women aged between 15 and 73 years, and between 14% to 24% of women of reproductive age present CPP. 2 In Brazil, the prevalence is not well known; international studies have demonstrated a high prevalence of persistent pain in Brazilian women, ∼ 36% in Rio de Janeiro, and 13% of women working in São Paulo. 3 In the city of Ribeirão Preto, the detected prevalence was of 11.5%, and 15.1% of these women are of reproductive age. 4

Endometriosis is among the gynecological causes related to CPP, and its main clinical problem is painful syndrome, manifesting as dysmenorrhea, pelvic pain, abdominal pain, dyspareunia, and painful defecation. 5 In women with CPP subjected to laparoscopy, the presence of endometriosis is higher than 30%. 2 The mechanisms involved are not clear yet, and the nature of the pain associated with endometriosis has been poorly characterized. Evidence suggests that the pain may be caused by peritoneal inflammation, formation of adherences, and significant nervous injury, specific to endometriosis injuries, which are possibly correlated with a deep infiltration of the endometrial tissue. 6

The scientific evidence of the relationship of overweight and obesity in women with CPP has not yet been clarified. The association between BMI and endometriosis has been studied; Viganò et al 7 concluded that women with endometriosis tend to be thinner than women without the disease, and the severity of the disease seems to be associated with the BMI. Yi et al 8 showed that women with advanced diseases had lower BMIs than those with minimal or mild diseases.

Endometriosis is closely associated with alterations of the immune and endocrine systems, 9 which are systems that are also associated with the control of obesity. To identify the etiological or mediating pathways associated with adiposity and endometriosis, one study 10 has suggested that the adipose tissue has immune properties. It is possible that these immune properties of the adipose tissue are involved or affected by the development of endometriosis, which can arise from an alteration in immune functioning. 9 The inhibition or promotion of immune cells may be linked to inflammation and to stimulation of angiogenesis. 12

Overweight has a high prevalence among females, and it is even higher during perimenopause: it may affect 60% of the women in this stage of life, probably due to metabolic alterations inherent to this period. It is also associated with bad eating habits and genetic predisposition. 13 The BMI and the percentage of body fat (%BF) may be involved in the relationship between overweight and obesity, which can influence the report of painful perception in women with CPP. Han et al 14 reported that an elevated waist-to-hip ratio (WHR) 15 indicates a pattern of central obesity, and it was significantly associated with chronic low-back pain in women, but not in men. Planning an effective treatment might help in the early identification of problems related to CPP secondary to endometriosis, and reducing and relieving the distress suffered by these women is of great importance. Therefore, the present study aimed to evaluate whether there is a difference in average body composition (%BF) through an evalution of the anthropometric markers of women diagnosed with CPP secondary to endometriosis and those diagnosed with CPP secondary to other causes. Moreover, we aimed to correlate the average body composition (%BF) with the intensity of the clinical pain in these women.

## Methods

The description of the present study was developed according to the Strengthening the Reporting of Observational Studies in Epidemiology (STROBE) 16 guidelines ( http://www.strobe-statement.org ).

The present observational case-control study was approved by the Ethics Committee of the teaching hospital at Faculdade de Medicina de Ribeirão Preto, Universidade de São Paulo (HCFMRP-USP), as well as its informed consent form (ICF). Women diagnosed with CPP at the outpatient clinic of pelvic pain were invited to participate in the study before starting any treatment.

The adopted inclusion criteria were women aged between 18 and 49 years, with CPP secondary to endometriosis or to other possible causes for at least 6 months, intense enough to interfere in routine activities, and demanding clinical or surgical treatment. All patients were submitted to laparoscopy and histology for the diagnosis of endometriosis. Women at menopause, those who were smokers, pregnant, and breastfeeding for the previous six months were excluded from the study, as well as those who refused to participate. Informed consent was obtained before the intervention and after the patients received explanations.

The patients who agreed to participate in the study were scheduled for an evaluation and advised to fast for 12 hours and to not change their eating behavior, 17 to not have products with caffeine: coffee, tea, and chocolate, 18 alcohol, 19 or medication 20 in the 24 hours preceding the interview, in which questionnaires were filled out and data were collected. Age, anthropometric data, (body mass, height), the perimeters of the arm, waist, abdomen, and hip, as well as the %BF were recorded.

To measure body mass and height, the women had to be barefoot, with no excess of clothes or accessories, with an erect body, the feet together, and the arms extended along the body, looking at a fixed point in the horizon. 21 Measurements were made using a Filizola (São Paulo, SP, Brazil) digital scale (0.1-kg scale), with a capacity for 150 kg with a coupled stadiometer. With the combination of body mass and height, we calculared the BMI (BMI = body mass/height 2 ). 22

To verify the perimeters of the arm, waist, abdomen, and hip, a Sanny (São Bernardo do Campo, SP, Brazil) non-elastic tape with 1 mm of precision was used. According to 23 the perimeter measurements, mainly the waist and hip perimeters are widely used, since they are fundamental to body composition.

The regional distribution of the %BF arouses concern by virtue of the association among health complications resulting from metabolic and cardiovascular dysfunctions and a larger accumulation of fat in the central region of the body.

The %BF was evaluated using the Model 310e. Bioimpedance Body Composition Monitor (Biodynamics Corporation, Shorelaine, WA, US). It sends a subthreshold electric current (800 µA, 50 kHz – single frequency), with gel electrodes (Hearbeat, Conmed corp., NY, USA) for bioimpedance. 24 It is a monitor that dysplays in a fast and precise way the amount of fat mass, lean mass, total body water, basal energy metabolism, and ideal weight. Its principle is based on electrical bioimpedamce, showing correlation levels, comparing to more accurate methods existing today. 25 To compare the %BF, we used values of ideal %BF according to Lohman, 26 classifying percentage from low (10% to 15%) to very high (> 30%).

To evaluate the intensity of the clinical pain, the visual analog scale (VAS) was used. 27 It consists of a 10-cm uninterrupted line in which the patient is instructed to check the score corresponding to the referred pain, keeping in mind that the beginning of the scale (0) corresponds to (no pain), and the end of the scale (10) corresponds to the “worst pain ever experienced or imagined.” For the classification of the pain, we considered mild pain: 5 mm to 44 mm; moderate pain: 45 mm to 74 mm; and severe pain: 75 mm to 100 mm. 28

The statistical analysis was performed using the 2016 version of the SAS University (SAS Institute, Inc., , Cary, NC, US) software, 29 and the results were shown as mean ± standard deviation or median and variation according to the distribution. At first, an exploratory data analysis was performed using the measurements of central position of dispersion and normality graphs. Given the asymmetry of the distribution of some variables, the non-parametric Mann Whitney test was used to compare the distribution of the quantitative variables in relation to the study groups.

## Results

**Fig. 1:**
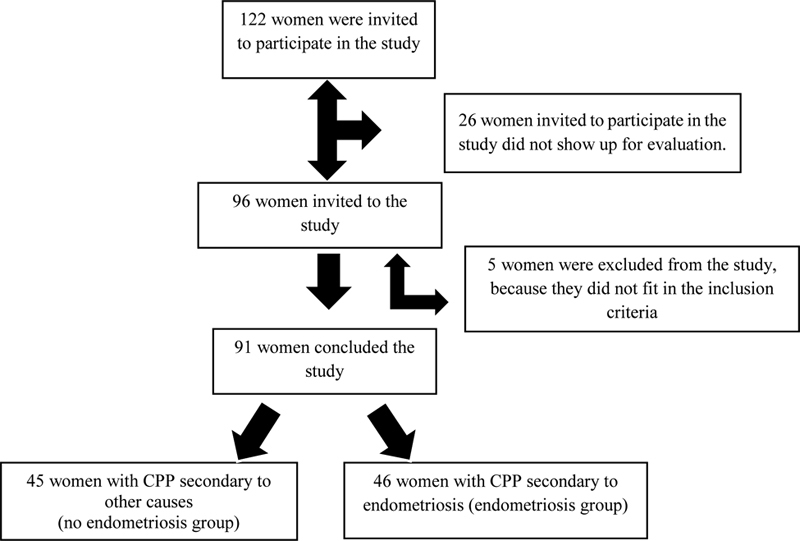
Recruitment of the evaluated women with and without endometriosis.

In total, 122 women clinically diagnosed with CPP were invited to participate in the study. Of them, 96 took part in the evaluation, 5 of them were excluded because they were at menopause, a period that interferes in the %BF, and 91 women remained: 45 were diagnosed with CPP secondary to other causes (the group was named CPP no endometriosis), and 46 women were diagnosed with CPP secondary to endometriosis (the group was named CPP endometriosis). The recruitment and evaluation took place from April 2014 to October 2015 ( Fig. 1 ).

Table 1 shows the description and comparison between both groups in relation to the demographic variables.

**Table 1:** Demographic characteristics of the women with CPP secondary to endometriosis and CPP secondary to other causes

Variables	Endometriosis ( *n* = 46)		No endometriosis ( *n* = 45)		
	Mean	SD	Mean	SD	*p* -value
**Age (years)**	36.78	± 7.58	38.33	± 7.42	0.32
****	**Mean**	**SD**	**Mean**	**SD**	***p*** -value
**Parity**	1.42	± 1.25	2.09	± 1.24	0.17
**Marital status**	**n**	**%**	**n**	**%**	***p*** -value
Married	31	68.9	34	73.91	0.59
Not married	14	31.11	12	26.09	
**Education**	**n**	**%**	**n**	**%**	***p*** -value
Primary	1	2.22	2	4.35	0.84
Elementary	16	35.56	15	32.6	
High School	19	42.22	22	47.83	
University	9	20	7	15.22	
**Profession**	**n**	**%**	**n**	**%**	***p*** -value
Working	30	65.91	31	68.89	0.76
Staying- at-home	15	34.09	15	31.11	

Abbreviations: CPP, chronic pelvic pain; SD, standard deviation.

Note: * *p* -values: non-parametric Mann Whitney test.

The comparison of the demographic variables (age, marital status, schooling, profession) between the two groups did not reveal a significant difference. It is important to notice that in both groups there is a high prevalence of overweight women or women with with grade-I obesity, whereas only 20% of both groups present a nutritional-status classification as eutrophic, in other words, appropriate according to the BMI. To classify the nutritional status of the women in both groups, the BMI and the %BF assessed by the electrical bioimpedance test were measured.

We observed that both groups had similar results regarding these variables, with no significant difference between them ( Table 2 ).

**Table 2:** Description of the nutritional status according to body mass index (BMI) and percentage of body fat of women with CPP secondary to endometriosis and CPP secondary to other causes evaluated through an electrical bioimpedance exam

		Endometriosis		No endometriosis		
**Classification**	** BMI (kg/m ^2^ ) **	**(** ***n*** ** = 45)**	**%**	**(** ***n*** ** = 46)**	**%**	***p*** **-value**
Eutrophic	18.5-24.9	9	20%	9	20%	0.9519
Overweight	25-29.9	18	40%	14	30%	
Obesity (grade I)	30-34.9	12	27%	10	22%	
Obesity (grade II)	35-39.9	2	4%	8	17%	
Obesity (grade III)	> 40	4	9%	5	11%	
**Classification**	**Percentage of body fat**	**(** ***n*** ** = 45)**	**%**	**(** ***n*** ** = 46)**	**%**	***p*** **-value**
Low	10-15%	0	0	0	0	0.0862
Great	12-25%	3	7%	2	4%	
Moderately high	25-30%	6	13%	6	13%	
High	30-35%	13	29%	11	24%	
Very high	> 30%	23	51%	27	59%	

Abbreviation: CPP, chronic pelvic pain.

Regarding the anthropometric variables presented in Table 3 (BMI, arm circumference, waist circumference, abdominal circumference, hip circumference, WHR and %BF), no difference among the women with CPP secondary to endometriosis was found.

**Table 3:** Description of anthropometric variables (BMI, arm perimeter, waist perimeter, abdominal perimeter, hip perimeter, waist-to-hip ratio and percentage of body fat) in women with CPP with endometriosis ( *n* = 45) and CPP with no endometriosis ( *n* = 46)

Anthropometric variables	Endometriosis (n = 45)		No endometriosis (n = 46)		*p* -value
	Mean	SD	Mean	SD	
BMI (Kg/m2)*	29.26	±6.23	30.84	±6.22	0.26
AbP (cm)	30.63	±4.37	31.06	±4.94	0.83
WP (cm)	88.04	±13.47	90.98	±14.36	0.24
AP (cm)	95.13	±13.67	98.39	±14.33	0.11
HP (cm)	106.4	±12.97	107.2	±12.71	0.45
WHR	0.83	±0.09	0.84	±0.76	0.19
Percentage of body fat	34.92	±6.1	36.01	±6.22	0.27

Abbreviations: AbP, abdominal perimeter; AP, arm perimeter; BMI, body mass index; HP, hip perimeter; WHR, waist-to-hip ratio; WP, waist perimeter.

Note: *p* -values: non-parametric Mann Whitney test.

In relation to the pain in the VAS, which was evaluated in both groups, there was a significant difference ( *p* < 0.05) between them ( Table 4 ).

**Table 4:** Description of the mean scores on the unidimensional scales for pain intensity – Visual Analog Scale (VAS) of women with or without endometriosis

Variables	*Endometriosis*		*No endometriosis*		
Visual Analog Scale	( *n* = 45)	Standard deviation	( *n* = 46)	Standard deviation	*p* -value
	7.2	±2.05	5.9	±2.62	0.03

Note: *p* -values: non-parametric Mann Whitney test.

## Discussion

In the present study, we performed an analysis of body composition using anthropometric markers, evaluating the %BF compared with the intensity of clinical pain in women with a clinical diagnosis of CPP secondary to endometriosis and secondary to other causes. The groups were homogeneous in relation to the number of women evaluated and showed no difference in relation to the analyzed parameters and body composition.

A study 7 demonstrated that women with endometriosis have physical characteristics of BMI below the classification that is considered for the healthy population: they are often thin, and do not present obesity in relation to the control subjects. Nevertheless, we observed in the present study that both groups showed a percentage of women with BMI above what is considered appropriate. However, the BMI does not necessarily represent an increase in adiposity, since it represents the total body mass, and not only adipose tissue mass, not reflecting the distribution of the %BF. 30

Gurian et al, 31 in their study, aimed to analyze anthropometric parameters (BMI and %BF), and the clinical and experimental pain in women with CPP; the study revealed that a large part of the evaluated women presented a very high %BF, in other words, risk of disease associated with obesity, 32 as shown in the present study.

According to and Ley et al, 33 the mean gain in body weight at perimenopause is estimated between 2 kg to 4 kg in 3 years, with an increase of 20% in total body fat. Therefore, as in the present study, the mean age of the women evaluated corresponds to the perimenopause phase, which can at least partly explain our results.

The %BF in women with CPP shown in the present study was above the health levels recommended by the World Health Organization (WHO). 23 Although there are few studies on body composition related to women with CPP and their secondary causes, 31 such as endometriosis, we can understand that this increased percentage is probably due to certain factors, such as sedentary lifestyle, eating habits, metabolic alterations, and genetic predisposition. 13

Several studies suggest that the adipose tissue has immune properties 10 and differs depending on the type and location of the tissue, 34 and that these properties may be involved or affected by the development of endometriosis. 9 Women with endometriosis particularly present small amounts of adipose tissue (body fat) and adipose tissue below the waist (WHR). In the study by Shah et al, 35 the waist circumference values showed a low relationship with endometriosis, and there was still no association between WHR and endometriosis. However, they suggest that a WHR below the recommended value may be related to up to a three-time higher chance of receiving the diagnosis of endometriosis. According to the present study, the WHR values between the groups did not show any difference, and this was the only ratio evaluated in the study.

Nevertheless, being thin is associated with the predominance of macrophages M2, whereas being overweight or obese is associated with the predominance of macrophages M1 (which promote inflammation and inhibit angiogenesis and tissue remodeling). 36 We observed in the present study that, despite the fact that both groups had CPP, the endometriosis group showed a high %BF, which was a finding that differed from those in the literature, and they also presented values higher than what is indicated for the region below the waist. Obesity is associated with significant increases in morbidity and mortality; obese patients are more prone to develop diseases, such as hypertension, dyslipidemia, type-II diabetes mellitus, coronary heart disease, cerebral vascular accident, pulmonary dysfunction, among other chronic diseases, as well aggaravation of the endometriosis. An in-depth investigation of the aspects of adiposity with endometriosis is necessary to identify etiological or mediating pathways between them. 7

Visscher et al 37 reported that the intensity of the pain has a great importance in the clinical practice, and it is recognized as one of the most relevant subjective pain experiences. The VAS is among the most widely used pain scales that aims to check the efficacy of the therapeutics and to compare the efficacy of different active treatments. It showed to be effective in the evaluation of the result of the treatments proposed in some randomized controlled trials applied to women with CPP of diverse causes; in them, the VAS was used as the primary outcome, as in the present study. 38

The main limitation of the present study is the women evaluated in the group without endometriosis who had different etiologies and comorbidities, which were not considered separately, but we do not believe that this compromised the results.

## Conclusion

We conclude that there was no difference in relation to body composition and the anthropometric parameters evaluated in both groups of patients with CPP (with or without endometriosis); however, the group of patients with endometriosis showed higher pain scores than the patients without endometriosis.
